# Effectiveness of the Interdisciplinary Home-bAsed Reablement Programme (I-HARP) on improving functional independence of people living with dementia: a multicentre, pragmatic, randomised, open-label, controlled trial

**DOI:** 10.1136/jnnp-2024-334514

**Published:** 2025-01-16

**Authors:** Yun-Hee Jeon, Judy Simpson, Judith Fethney, Luisa Krein, Mirim Shin, Lee-Fay Low, Robert T Woods, Loren Mowszowski, Sarah Hilmer, Sharon L Naismith, Lindy Clemson, Henry Brodaty, Vasi Naganathan, Amanda Miller Amberber, Danelle Kenny, Laura Gitlin, Sarah Szanton

**Affiliations:** 1 Susan Wakil School of Nursing and Midwifery, Faculty of Medicine and Health, The University of Sydney, Sydney, New South Wales, Australia; 2 School of Public Health, Faculty of Medicine and Health, University of Sydney, Sydney, New South Wales, Australia; 3 School of Health Sciences, Faculty of Medicine and Health, The University of Sydney, Sydney, New South Wales, Australia; 4 DSDC Wales, School of Health Sciences, Bangor University, Bangor, UK; 5 Brain and Mind Centre and School of Psychology, Faculty of Science, The University of Sydney, Sydney, New South Wales, Australia; 6 Kolling Institute and Faculty of Medicine and Health, Northern Sydney Local Health District and The University of Sydney, Sydney, New South Wales, Australia; 7 Centre for Healthy Brain Ageing, School of Psychiatry, UNSW Sydney, Sydney, New South Wales, Australia; 8 Concord Clinical School, Faculty of Medicine and Health, University of Sydney, Sydney, New South Wales, Australia; 9 Macquarie University, Sydney, New South Wales, Australia; 10 Centre for Health Services Research, The University of Queensland, Brisbane, Queensland, Australia; 11 College of Nursing and Health Professions, Drexel University, Philadelphia, Pennsylvania, USA; 12 Schools of Nursing, Medicine and Public Health, Johns Hopkins University, Baltimore, Maryland, USA

**Keywords:** DEMENTIA, REHABILITATION

## Abstract

**Background:**

We investigated the effectiveness of an Interdisciplinary Home-bAsed Reablement Programme (I-HARP) on improving functional independence, health and well-being of people with dementia, family carer outcomes and costs.

**Method:**

A multicentre pragmatic parallel-arm randomised controlled trial compared I-HARP to usual care in community-dwelling people with mild to moderate dementia and their family carers in Sydney, Australia (2018–2022). I-HARP is a 4-month, home-based, dementia rehabilitation model delivered by an interdisciplinary team. Assessments were conducted at baseline (time-1), 4-month (time-2) and 12-month (time-3) follow-up. The primary outcome measure was the client’s functional independence using the Disability Assessment for Dementia (DAD) scale at time-2, based on intention-to-treat analyses.

**Result:**

Of 130 recruited client-carer dyads, 116 dyads (58/group) completed the trial. The I-HARP group were not significantly better in most outcome measures than usual care at both time-2 and time-3; with the only statistically significant difference being a reduction in home environment hazards at time-2. Post hoc subgroup analysis of 66 clients with mild dementia found significantly better functional independence in the intervention group compared with those in usual care: difference 8.99 on DAD (95% CI 1.21, 16.79) at time-2 and difference 12.16 (95% CI 1.93, 22.38) at time-3. Economic evaluation suggests potentially lower resource use in I-HARP compared with usual care, but the cost-effectiveness is uncertain.

**Conclusion:**

Primary outcomes were not met for a population of people with dementia, with severity ranging from mild to moderate and severe. The I-HARP model appeared to benefit functional independence of participants with mild dementia, with potential cost savings.

**Trial registration number:**

ACTRN12618000600246.

**WHAT IS ALREADY KNOWN ON THIS TOPIC**

**WHAT THIS STUDY ADDS**

- An interdisciplinary rehabilitation model of care for community-dwelling people with dementia and their family carers can be effective in improving and sustaining the independence of people with mild dementia but not for those with moderate to severe dementia.

**HOW THIS STUDY MIGHT AFFECT RESEARCH, PRACTICE OR POLICY**

- The provision of an early and timely reablement model of care led by interdisciplinary teamwork that considers the person’s physical, medical, psychological and social care needs should be a key part of postdiagnostic services for people with dementia and their carers.

## Introduction

 People with dementia can improve and/or maintain their functional independence through accessing appropriate rehabilitation interventions.^1 2^ WHO defines rehabilitation as a suite of interventions that are used to address a broad range of functional losses or declines through prevention, risk minimisation, maintenance, restoration and/or improvement of function, as well as compensation for lost function.^3 4^

Approximately one in three people with a health condition can benefit from rehabilitation at some point in their illness trajectory, and dementia is no exception.^5^ A systematic review of six quality clinical practice guidelines for dementia^1^ identified many rehabilitation-related interventions suitable for routine practice; however, attention to implementation processes for these interventions is lacking. Similarly, a review of dementia rehabilitation programmes that usually combine more than one rehabilitation intervention for dementia^2^ indicates growing evidence of positive effects, and yet little is known as to how they can be feasibly, cost-effectively and routinely implemented as part of comprehensive dementia care.

This lack of implementation research outcomes for dementia rehabilitation is particularly problematic as most of the dementia intervention trials do not account for chronic and complex care needs attributable to physical and medical comorbidities and multimorbidity commonly experienced by older people with dementia.^6^,^8^ A comprehensive model of care with appropriate staffing and resources is needed to address these complexities in everyday clinical environments.

The US’s Community Aging in Place Advancing Better Living for Elders (CAPABLE) program^9^,^11^ addressed these gaps in rehabilitation and demonstrated improvement in self-care abilities and independence of frail older people who often experience environmental risks for disability, functional decline and multimorbidity. Building on CAPABLE, the Australian model, Interdisciplinary Home-bAsed Reablement Programme (I-HARP), is specifically designed for those with cognitive impairment and dementia. I-HARP integrates proven strategies into a comprehensive, person-centred, home-based, interdisciplinary intervention integrated with existing community-based services. Its goal is to enhance the day-to-day function of older persons with dementia and other comorbid chronic age-related conditions.^12 13^

Our pilot randomised controlled trial (RCT) of I-HARP^13^ showed adequacy and appropriateness of the study design and procedures for randomisation, screening, recruitment and consent, and adherence to the programme. Compared with ‘usual care’, the I-HARP pilot suggested positive results for goal attainment, mobility and independence, continued living at home with no entry to higher care and self-perceived and observed client’s well-being and confidence.^13^

Our current study aimed to evaluate effectiveness of the I-HARP model on functional independence, mobility, quality of life and depression among people with dementia, their home environmental safety, carer burden and quality of life and its cost-effectiveness of the intervention. We also explored implementation aspects of the I-HARP model in hospital outreach and aged care services, which will be reported separately.

## Method

The study tested the following hypotheses.

Four months after baseline (at time-2), compared with the usual care group, the intervention group will have:

- Improved functional independence (primary outcome).
- Enhanced quality of life.
- Improved mobility.
- Reduced depressive symptoms.
- Improved carer quality of life.
- Decreased carer burden.
- Improved home environment safety.

12 months after baseline (at time-3), compared with the usual care group, the intervention group will have:

- Sustained the benefits of the intervention (1–7 above).
- Decreased total healthcare costs.

The study protocol with intervention details and analysis plan for this multicentre pragmatic parallel-arm stratified RCT provides methodological details.^12^

### Sites and participants

Client participants (clients hereafter) were recruited from three large public hospitals during specialist geriatrician outpatient appointments and from two community aged care providers in Sydney, New South Wales, Australia. Eligibility was determined at the time of screening and following consent before the baseline assessment. To be eligible, clients with dementia had to be recipients of healthcare or care services from participating sites, have mild to moderate dementia rated on the Global Deterioration Rating Scale for Assessment of Primary Degenerative Dementia (GDRS)^14^ stages 4–5 mild-moderate, be aged 60 years or over, have conversational English and have a cognitively able carer with at least 4 days or 7 hours per week contact. Clients with dementia were ineligible if they had a terminal illness with <1 year expected survival; were having active cancer therapy; planned to move in<1 year; were on a cholinesterase inhibitor but not on a stable dose for at least 3 months or had severe dementia (GDRS 6–7). Carer participants (carers hereafter) were deemed ineligible if they had moderate to severe cognitive impairment.

### Study procedures

Data were collected at baseline/time-1 (preintervention), time-2 (4 months postbaseline, after the intervention) and time-3 (12 months postbaseline) between May 2018 and March 2022. Intervention duration and follow-up time points were determined based on previous trials.^10 11 13 15^ We allowed for 2 months of ‘stop-the-clock’ rule whereby the intervention and/or assessment time was adjusted following events (eg, hospital admission, respite, pandemic lockdown and holiday), resulting in a longer than planned interval between time-1 and time-2 assessments (on average 22.5 weeks for control and 28 weeks for intervention). All assessments were conducted by experienced clinicians blinded to group allocation.

**Figure 1:**
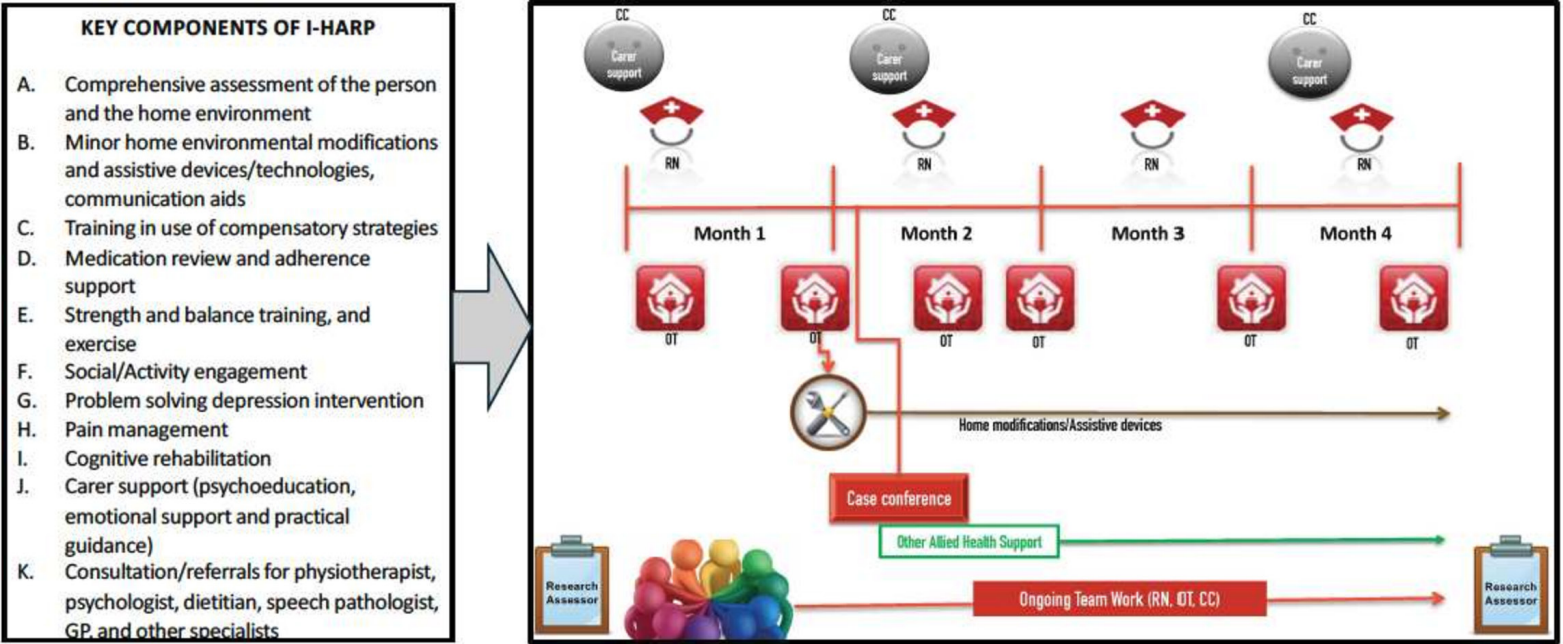
Key components of I-HARP and a pictorial illustration of the I-HARP delivery (adapted version^12^). I-HARP, Interdisciplinary Home-bAsed Reablement Programme. CC: Case Coordinator, OT Occupational Therapist, RN: Registered Nurse.

As shown figure 1, the intervention group received usual care plus I-HARP over 4–6 months including up to six home visits by an occupational therapist (OT), up to four visits by a registered nurse (RN) and three carer support sessions. Care was delivered using an interdisciplinary, person-centred and reablement-focused approach and facilitated by a case conference and regular team email/telephone follow-ups, based on the detailed step-by-step I-HARP intervention protocol. Additionally, clients were eligible for up to four other allied health sessions and minor home modifications and/or assistive devices (valued up to $A1000). The control group received usual care from their hospital or community-aged care service, which could have included ad hoc nursing and/or allied health and home modifications. During the COVID-19 pandemic, following a brief cessation, ethics approval was gained to resume the intervention for existing clients as it was deemed an important part of care. However, recruitment and enrolment of new participants were severely impacted by the pandemic (online supplemental eMethods for details).

### Outcome measures

Outcome measures were administered by trained clinical assessors. The primary outcome was clients’ functional independence at time-2, assessed using the Disability Assessment for Dementia (DAD), a 40-item dementia specific functional independence tool, which measures self-care, disability and independent living skills, completed by carers as a proxy.^16^ DAD was designed for people with dementia (17 items for basic self-care; 23 items for instrumental activities of daily living). The total converted percentage ranges from 1 to 100, with higher scores indicating greater independence.

Secondary outcomes were (1) for clients, functional independence at time-3; short-term and long-term effects on mobility and physical function (the Short Physical Performance Battery^17^), depression (the Collateral Source Geriatric Depression Scale-15 item^18^), quality of life (the Quality of life in Alzheimer’s disease^19^), health-related quality of life (the 5-level EQ-5D version,^20^ EQ-5D-5L) and home environmental safety (the Home Safety Self-Assessment Tool,^21^ HSSAT); (2) for carers, carer burden (the Zarit Burden Inventory^22^) and carer quality of life (EQ-5D-5L) and (3) total healthcare costs over 12 months following randomisation including clients’ use of prescribed medicines and associated costs, healthcare and community services (type, duration, frequency and personal costs), obtained through monthly telephone contact and a carers’ diary/log. We also collected detailed incidents of hospital transfers, falls and minor injuries during monthly phone calls.

At baseline, the clinical assessors collected sociodemographic data (age, sex, ethnicity, pension status, health insurance and education) and clinical data (health, medications and cognitive conditions using the Addenbrooke’s Cognitive Examination Third edition,^23^ ACE-III). Severity of dementia was assessed with GDRS (range 1–7) with higher scores associated with more severe dementia.^14^ Scores were dichotomised to mild dementia severity (GDRS 4) or moderate to severe (GDRS≥5) for subgroup analysis.

### Sample size

We used pilot results^13^ to calculate sample size. As per the pilot, to detect a medium (Cohen’s d=0.5) intervention effect size in the primary outcome (DAD) between the two groups at time-2 (4 months, short-term effect) and time-3 (12 months, longer-term effect), with 5% two-sided significance and 80% power, a total of 64 dyads (128 total dyads) per group were required. The target sample size was increased to 170 total dyads to allow for up to 25% attrition. A medium effect size equates to approximately a 6-point difference (SD 10.65) implying ability to carry out six more activities assessed by the DAD in one group compared with the other.^13^

### Randomisation and masking

Participants (both client and carer) were randomised following baseline assessment (time-1) to either the intervention or control arm (1:1). Randomisation was performed separately by an independent research assistant for each hospital/aged care site and stratified by severity of dementia (mild vs moderate), using computer-generated random permuted blocks of varying size. Opaque sequentially numbered envelopes ensured allocation concealment. Participants were not blinded to group allocation due to the nature of the intervention, but the clinical assessors, statisticians and research assistants involved in data collection and analysis were blinded to group allocation. Participants were notified of group allocation by phone and the importance of the blinding process and the need to maintain blinding was explained.

### Data analysis

Analyses of all outcomes were by intention to treat. Comparison of intervention and control groups at time-2 (short-term effect) and time-3 (longer-term effect) used the analysis of covariance (ANCOVA) approach to adjust for the baseline value of that outcome, stratification variables (site and severity of dementia) and other covariates (age and pension status). Falls, hospital transfers and admissions were analysed using negative binomial or Poisson regression based on the dispersion of the data. IBM SPSS Statistics V.28 and R were used for all analyses. P values <0.05 were considered statistically significant. No adjustment was made for multiple comparisons because all analyses were prespecified unless noted to be post hoc. There were 8.2% missing data across the entire data set. Missing data were imputed using the Markov Chain Monte Carlo algorithm with predictive mean matching using 5 nearest neighbours and 20 imputations. See online supplemental eMethods for management of missing data and detailed data analyses.

The economic evaluation considered the intervention cost (ie, clinicians’ time and expense to deliver I-HARP, travel, care coordination time, costs for minor home modification/assistive devices and training) and overall health-related client costs (medications, visits to specialists, GPs, hospitals, residential aged care (RAC) admission) over the 52-week period. A cost–utility analysis combined these cost data with health-related quality of life (EuroQol 5-Dimension 5-Level, EQ-5D-5L), reporting a ‘within-trial result’, a cost per quality-adjusted life-year (QALY) of I-HARP relative to control over 52 weeks. We used non-parametric bootstrapping to achieve a more robust incremental cost-effectiveness ratio (ICER) estimate while accommodating the skew in both cost and QALY,^24^ using the boot package in RStudio.^25^ A detailed cost calculation for all items, along with the data analyses, has been reported in full.^26^

As clients had either mild (GDRS 4) or moderate-severe dementia (GDRS≥5), we performed a post hoc subgroup analysis for the DAD by baseline dementia severity category.

## Results

### Participant flow and baseline characteristics

**Figure 2:**
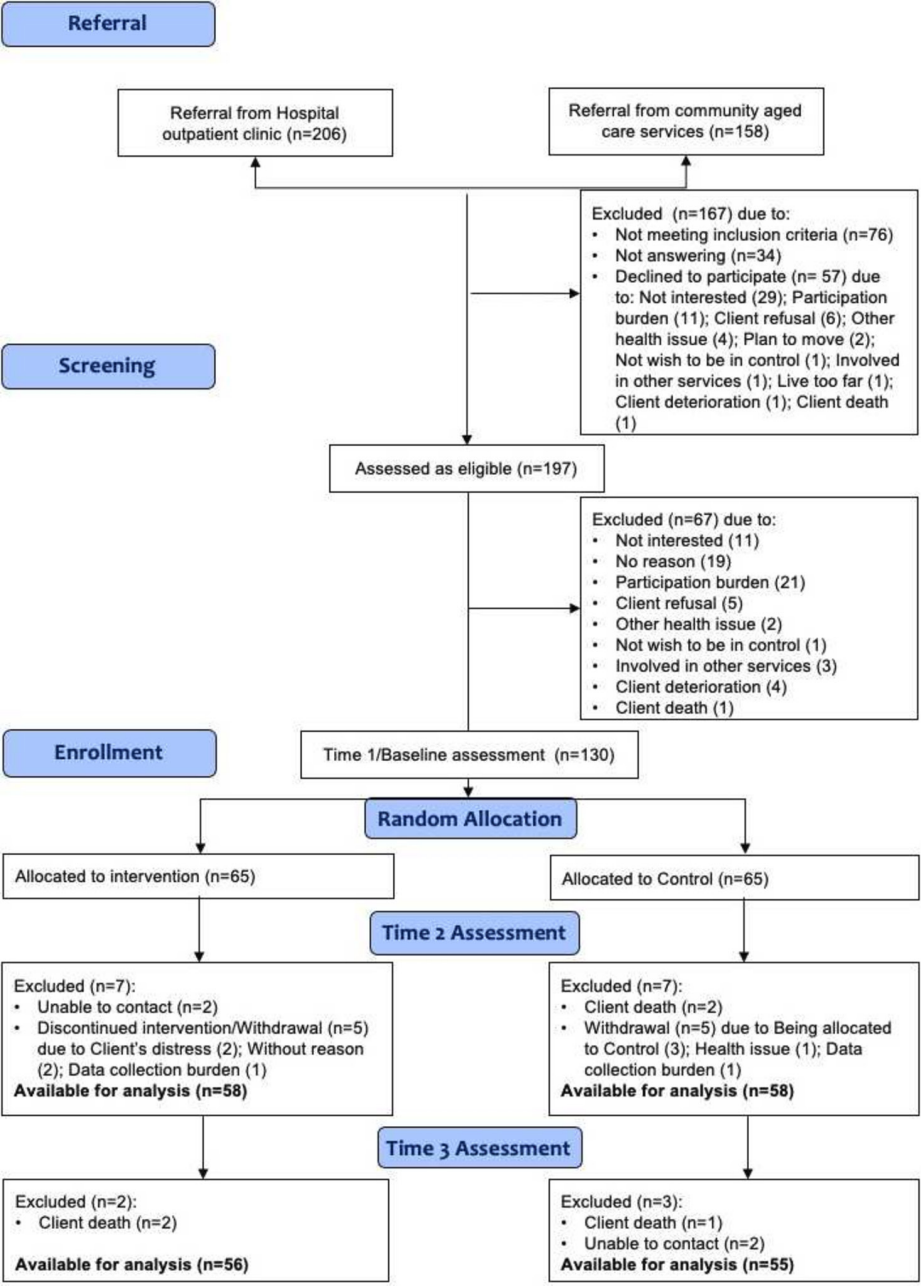
Flow of participants through trial.

Across the 6 sites, 130 dyads were enrolled: 24/130 dyads enrolled post-COVID-19, between April 2020 and March 2021. Study attrition was low, with 14 dyads (11%) lost by time-2; 10 dyads withdrew, 2 dyads were unable to contact and 2 clients died, leaving 116 dyads (58 per group) who continued to time-2 (figure 2). Baseline comparison of dyads who withdrew versus continued is reported in online supplemental table 1. The only statistically significant difference was for the Carer EQ-5D-5L, where those who discontinued had a lower mean score (0.75 vs 0.61: 95% CI 0.002, 0.27, p=0.047). Clients who entered RAC post-enrolment and their carers remained in the study. Between time-2 and time-3, two clients withdrew and three died, leaving 111 dyads available for analysis at time-3 (85.5% retention overall).

Table 1 shows the baseline characteristics of the 58 dyads in each group. Clients had a mean age of 81.4 (SD 6.70) years and 57% had mild dementia, with a mean ACE-III score of 53.9 (SD 19.87). Most were female (63%), 90% spoke English as a main language, 47% had less than 10 years of education and 75% were on pensions. Carers were a mean age of 66.3 (SD 13.64) years, mostly female (68%), spoke English at home (93%) and 21% had less than 10 years of education.

**Table 1:** Demographics of I-HARP participants at baseline

	Control N=58	Intervention N=58
	n (%)	n (%)
**Person with dementia (client)**		
Age in years, mean (SD) (range)	81.6 (6.97) (68– 97)	81.2(6.46) (60–94)
Sex, n (%)		
Male	26 (45)	17 (29)
Female	32 (55)	41 (71)
Marital status*		
Single	1 (2)	1 (2)
Married/de facto	33 (57)	37 (64)
Separated/divorced	6 (10)	5 (9)
Widowed	16 (28)	15 (26)
Education†		
<10 years education	23 (40)	32 (55)
Leaving certificate/higher school certificate	8 (14)	9 (16)
Technical and further education/vocational	8 (14)	9 (16)
University	18 (31)	7 (12)
English main language at home (yes)*	50 (86)	54 (93)
Country of birth (Australia)	36 (62)	36 (62)
Private health insurance (yes)‡	39 (67)	41 (71)
Receives home care or community services§	38 (66)	35 (60)
Global Deterioration Rating Scale for (GDRS) stages		
Stage 4: mild dementia	34 (59)	32 (55)
Stage 5: moderate dementia	19 (33)	21 (36)
Stage 6: severe cognitive decline¶	5 (9)	5 (9)
Addenbrooke’s Cognitive Examination III, mean (range)	54.83 (5–92)	53.07 (5–91)
Referral through		
Hospital	29 (50)	33 (57)
Aged care	29 (50)	25 (43)
Pension (aged care pension and/or seniors healthcare card)	45 (78)	42 (72)
**Carer**		
Age in years, mean (SD) (range)	66.5 (13.52) (38–89)	66.0 (13.88) (33–93)
Sex, n (%)		
Male	17 (29)	20 (35)
Female	41 (71)	38 (66)
Education		
<10 years education	12 (21)	12 (21)
Leaving certificate/higher school certificate	8 (14)	8 (14)
Technical and further education/vocational	12 (21)	19 (33)
University	26 (45)	19 (33)
Current work status		
Full time	7 (12)	7 (12)
Part time	9 (15)	6 (10)
Other income-generating	5 (5)	7 (12)
No paid employment/fully retired	37** (64)	38†† (66)
English main language at home (yes)†	53 (91)	55 (95)
Country of birth (Australia)	44 (76)	41 (71)
Private health insurance (yes)	48 (83)	44 (76)
Pension (aged care pension and/or seniors healthcare card)	20 (35)	24 (41)
Hours per day caring, mean (range)	14.3 (0.25–24)	12.08 (1.0–24)
Relationship of carer to client‡		
Spouse/partner	30 (52)	30 (52)
Adult children	20 (34)	22 (38)
Others	7‡‡ (12)	6§§ (9)
Anyone else involved in caring for the client (yes)	23 (40.0)	31 (53)
Weekly caring hours by additional carers, mean (range)	13.3 (1–50)	10 (1–52)

* 2 observations missing for control.

† 1 observation missing from each group.

‡ 1 observation missing for control.

§ 3 observations missing for control.

¶ 3 clients in control and 4 in intervention with GDRS stage 5 or 6 unsure were grouped into stage 6. Those noted as having a GDRS of 6 at baseline had a GDRS of 5 at screening.

** Control: 31×fully retired, 1×unpaid work, 1×studying, 3×home duties, 1×unemployed.

†† Intervention: 27×fully retired, 7×home duties; 2×unemployed; 2×unpaid work.

‡‡ 2×client siblings; 3×daughter-in-law; 1×friend; 1×nephew.

§§ 2×client siblings; 1×daughter-in-law; 1×son-in-law; 2×unspecified.

I-HARP, Interdisciplinary Home-bAsed Reablement Programme.

### Effectiveness: client, carer and environment-related outcomes

Results are based on multiply imputed data. Unadjusted means and change from baseline to time-2 and time-3 are reported, while the mean differences and the associated 95% CIs are based on adjusted analyses. Results for all outcomes from baseline to time-2 are reported in table 2. There was no statistically significant effect for the primary outcome, client independence measured by the DAD. Client independence decreased (worsened) in both groups, although the intervention group decreased less, with the adjusted mean score of the intervention group 3.22 points higher (95% CI −3.34, 9.79, p=0.34) than the control group.

**Table 2:** Outcomes baseline/time-1 (T-1) to time-2 (T-2) and time-3 (T-3)

Outcomes	Control					Intervention					Adjusted difference	
	T-1 Mean	T-2 Mean	T-3 Mean	Change at T-2	Change at T-3	T-1 Mean	T-2 Mean	T-3 Mean	Change at T-2	Change at T-3	Between T-1 to T-2 intervention vs control* (95% CI), p value	Between T-1 to T-3 intervention vs control* (95% CI), p value
Client outcomes												
DAD	60.76	51.05	44.34	−9.71	−16.42	58.89	52.72	45.98	−6.17	−12.91	3.22 (−3.34, 9.79), 0.34†	2.57 (−5.17, 10.31), 0.52†
CS-GDS-15	6.69	6.32	7.64	−0.37	0.95	7.69	7.26	7.86	−0.43	0.17	0.30 (−0.88, 1.49), 0.62†	−0.27 (−1.76, 1.21), 0.72†
QoL-AD Client	37.23	36.13	34.37	−1.10	−2.86	36.82	36.59	36.01	−0.23	−0.81	0.65 (−1.18, 2.48), 0.49†	1.82 (−1.82, 5.46), 0.32†
QoL-AD Proxy	29.95	30.18	29.27	0.23	−0.68	30.80	31.33	30.43	0.53	−0.37	0.70 (−1.00, 2.41), 0.42†	0.64 (−1.54, 2.81), 0.57†
EQ-5D-5L	0.78	0.78	0.70	−0.002	−0.08	0.78	0.83	0.74	0.05	−0.03	0.05 (−0.04, 0.13), 0.28†	0.05 (−0.05, 0.15), 0.29†
EQ-5D-5L VAS	73.67	75.76	69.31	2.09	−4.36	71.68	70.66	69.27	−1.02	−2.41	−4.46 (−11.61, 2.69), 0.22†	0.60 (−8.47, 9.67), 0.89†
SPPB	6.82	6.03	4.85	−0.80	−1.97	6.76	5.93	5.75	−0.83	−1.01	−0.06 (−1.01, 0.89), 0.90†	0.92 (−0.35, 2.20), 0.15†
Environment outcome												
HSSAT	14.69	12.72	13.06	−1.97	−1.63	15.53	11.27	12.14	−4.26	−3.39	−2.06 (−3.67,–0.46), 0.01†	−1.17 (−5.73, 3.38), 0.61†
Carer outcomes												
ZBI	31.69	32.61	35.58	0.92	3.89	30.37	31.77	32.41	1.40	2.04	0.23 (−3.05, 3.52), 0.89‡	−2.06 (−7.55, 3.44), 0.46‡
EQ-5D-5L	0.74	0.71	0.68	−0.03	−0.06	0.75	0.67	0.73	−0.08	−0.02	−0.04 (−0.12, 0.04), 0.35‡	0.04 (−0.05, 0.14), 0.36‡
EQ-5D-5L VAS	76.03	76.05	68.94	0.02	−7.09	75.84	72.92	72.38	−2.92	−3.46	−2.79 (−8.48, 2.90), 0.34‡	3.65 (−4.01,11.30), 0.35‡

* Difference indicates how much higher (if positive) or lower (if negative) Intervention mean score is versus control mean score in adjusted analysis.

† Adjusted for site, dementia severity, client pension, client age and baseline score of the outcome.

‡ Adjusted for site, dementia severity, carer age, carer pension and baseline score.

CS-GDS-15, Collateral Source version of the Geriatric Depression Scale-15 item; DAD, Disability Assessment for Dementia; EQ-5D-5L, EuroQol 5-Dimension 5-Level; HSSAT, Home Safety Self-Assessment Tool; QoL-AD, Quality of life in Alzheimer’s disease; SPPB, Short Physical Performance Battery; VAS, Visual Analogue Scale; ZBI, Zarit Burden Inventory.

Among the secondary outcomes, the HSSAT for home environmental safety at time-2 was the only outcome with a statistically significant difference, with adjusted mean score 2.06 points lower (fewer hazards) in the Intervention (95% CI 0.46, 3.67, p=0.01). As some clients entered RAC by time-2 (two control and five intervention clients), the HSSAT score for those who did not enter RAC was separately analysed. The significant difference remained, with control reducing by 1.74 hazards and intervention by 3.67 hazards, adjusted difference 1.93 (95% CI 0.19, 3.67, p=0.03). For the carer outcomes, there were no statistically significant differences at time-2.

By time-3, there were no statistically significant differences for any client or carer outcomes.

### Effectiveness: subgroup analysis by dementia severity

**Figure 3:**
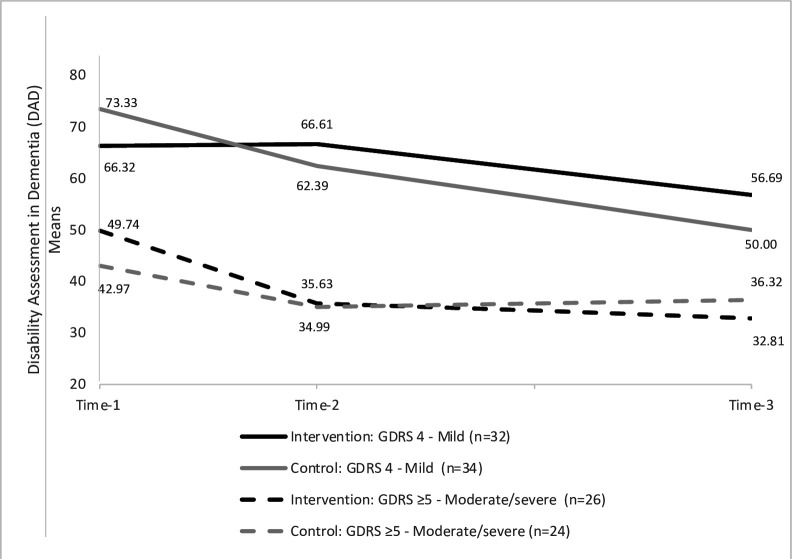
Self-care ability and independent living skills stratified by severity of dementia at baseline. Global Deterioration Rating Scale for Assessment of Primary Degenerative Dementia (GDRS); Time-2 is 4 months from baseline/Time-1 and Time-3 is 12 months from Time-1.

Table 3 shows adjusted post hoc comparisons of all outcomes by group and dementia severity category. Mean scores over time are shown in figure 3 to indicate the trend. At time-2, in the mild dementia group intervention mean DAD scores were higher (greater independence) than controls (adjusted difference 8.99, 95% CI 1.21, 16.79, medium effect size), while in the moderate-severe group, there was no difference (adjusted difference −4.39, 95% CI −15.61, 6.83).

**Table 3:** Outcomes baseline/time-1 (T-1) to time-2 (T-2, 4 months) and time-3 (T-3, 12 months) split by dementia severity

Outcomes	Control: stage 4 n=34; stage ≥5 n=24					Intervention: stage 4 n=32; stage ≥5 n=26					Adjusted difference	
	T-1 Mean	T-2 Mean	T-3 Mean	Change at T-2	Change at T-3	T-1 Mean	T-2 Mean	T-3 Mean	Change at T-2	Change at T-3	Between T-1 to T-2 intervention vs control* (95% CI)	Between T-1 to T-3 intervention vs control* (95% CI)
Client outcomes												
St 4: DAD	**73.33**	**62.39**	**50.00**	**−10.94**	**−23.33**	**66.32**	**66.61**	**56.69**	**0.29**	**−9.64**	**8.99 (1.21, 16.79)†‡**	**12.16 (1.93, 22.38)‡§**
St≥5: DAD	42.97	34.99	36.32	−7.98	−6.65	49.74	35.63	32.81	−14.11	−16.93	−4.39 (−15.61, 6.83)‡	−7.16 (−18.78, 4.46)‡
St 4: CS-GDS-15	5.77	5.82	7.52	0.05	1.75	6.35	6.14	7.11	−0.21	0.76	−0.10 (−1.47, 1.28)‡	−0.87 (−2.741.01)‡
St≥5: CS-GDS-15	8.01	7.02	7.82	−0.99	−0.19	9.34	8.63	8.77	−0.71	−0.57	0.66 (−1.39, 2.70)‡	0.69 (−1.77, 3.14)‡
St 4: QoL-AD-S	37.52	35.74	35.36	−1.78	−2.16	36.28	36.48	36.16	0.20	−0.12	1.77 (−0.44, 3.97)‡	1.66 (−2.32, 5.65)‡
St≥5: QoL-AD-S	36.81	36.69	32.96	−0.12	−3.85	37.49	36.71	35.82	−0.78	−1.66	−0.40 (−3.69, 2.90)‡	2.14 (−3.79, 8.06)‡
St 4: QoL-AD - P	31.83	31.15	30.05	−0.69	−1.78	31.97	32.62	31.65	0.65	−0.32	1.53 (−0.63, 3.63)‡	1.43 (−1.43, 4.29)‡
St≥5: QoL-AD-P	27.29	28.81	28.16	1.52	0.87	29.36	29.73	28.93	0.38	−0.43	0.41 (−2.25, 3.07)‡	−0.78 (−4.20, 2.64)‡
St 4: EQ5D5L	0.79	0.80	0.72	0.01	−0.06	0.71	0.79	0.74	0.08	0.03	0.05 (−0.06, 0.15)‡	0.04 (−0.09, 0.17)‡
St≥5: EQ-5D-5L	0.77	0.75	0.66	−0.02	−0.11	0.86	0.86	0.78	0.007	−0.08	0.06 (−0.08, 0.20)‡	0.07 (−0.08, 0.22)‡
St 4: EQ VAS	77.25	76.36	71.17	−0.89	−6.08	72.96	71.37	72.24	−1.59	−0.72	−3.88 (−12.02, 4.26)‡	3.43 (−6.72, 13.58)‡
St≥5: EQ VAS	68.59	74.91	66.68	6.32	−1.90	70.11	69.79	65.61	−0.32	−4.49	−5.82 (−18.69, 7.05)‡	−2.76 (−18.39, 12.88)‡
St 4: SPPB	7.74	6.75	5.46	−0.99	−2.28	6.75	6.44	5.90	−0.31	−0.85	0.51 (−0.80, 1.83)‡	1.05 (−0.64, 2.73)‡
St≥5: SPPB	5.54	5.00	3.99	−0.54	−1.55	6.77	5.30	5.58	−1.47	−1.19	−0.64 (−2.08, 0.81)‡	0.96 (−1.27, 3.20)‡
Environment outcome												
St 4: HSSAT	14.59	12.96	13.32	−1.63	−1.27	14.69	11.81	12.60	−2.87	−2.09	−1.49 (−3.35, 0.37)‡	−0.45 (−5.53, 4.63)‡
St≥5: HSSAT	**14.83**	**12.38**	12.70	**−2.45**	−2.13	**16.58**	**10.60**	11.57	**−5.98**	−5.01	**−3.14 (−6.10, to 0.17)‡**	−0.51 (−8.50, 7.47)‡
Carer outcomes												
St 4: ZBI	27.80	30.38	33.63	2.58	5.82	27.42	28.85	30.13	1.43	2.71	−0.99 (−5.18, 3.20)¶	−1.77 (−7.63, 4.08)¶
St≥5: ZBI	37.20	35.77	38.35	−1.43	1.15	34.00	35.36	35.23	1.36	1.22	1.61 (−3.71, 86.93)¶	−1.82 (−11.74, 108.10)¶
St 4: EQ-5D-5L	0.75	0.74	0.73	−0.01	−0.02	0.74	0.69	0.74	−0.05	0.004	−0.04 (−0.15, 0.07)¶	0.02 (−0.10, 0.14)¶
St≥5: EQ-5D-5L	0.73	0.66	0.62	−0.07	−0.11	0.75	0.65	0.71	−0.10	−0.04	−0.03 (−0.16, 0.10)¶	0.07 (−0.09, 0.23)¶
St 4: EQ VAS	75.56	77.12	72.50	1.56	−3.06	76.34	75.13	75.65	−1.21	−0.68	−1.47 (−8.78, 5.84)¶	3.13 (−5.73, 11.98)¶
St≥5: EQ VAS	76.70	74.54	63.90	−2.16	−12.80	75.23	70.19	68.36	−5.04	−6.87	−3.57 (−12.90, 75.77)¶	5.00 (−8.78, 18.79)¶

* Difference indicates how much higher (if positive) or lower (if negative) intervention mean score is versus control mean score in adjusted analysis.

† Effect size 0.08.

‡ Adjusted for site, client pension, client age and baseline score of the outcome.

§ Effect size 0.09 (partial eta2 0.01 (small); 0.06 (medium); 0.14 (large).^40^

¶ Adjusted for site, carer age, carer pension and baseline score.

CS-GDS-15, Collateral Source version of the Geriatric Depression Scale-15 item; DAD, Disability Assessment for Dementia; EQ-5D-5L, EuroQol 5-Dimension 5-Level; HSSAT, Home Safety Self-Assessment Tool; QoL-AD, Quality of life in Alzheimer’s disease; SPPB, Short Physical Performance Battery; VAS, Visual Analogue Scale; ZBI, Zarit Burden Inventory.

At time-3 in the mild dementia group, intervention mean DAD scores were again higher than controls (adjusted difference 12.16, 95% CI 1.93, 22.38, medium effect size), while in the moderate-severe group, there was no difference (adjusted difference −7.16, 95% CI −18.78, 4.46).

### Other outcomes: falls and hospital and RAC use

#### Falls

At time-2, there were 46 total falls in the intervention group and 81 falls in the control group experienced by 21/58 clients for each group. In an adjusted analysis using negative binomial regression, the relative rate of total falls for intervention versus control was 0.54 (95% CI 0.25, 1.19, p=0.13, mean falls 0.76 vs 1.41). At time-3, there was a statistically significant intervention effect. While both groups reported an increase in total falls at time-3, there were 93 total falls experienced by 33/55 intervention clients, and 252 falls experienced by 33/56 control clients, giving a relative rate of 0.40 (95% CI 0.21, 0.75, p=0.005, mean falls 1.76 vs 4.42) total falls in the intervention versus control groups (online supplemental table 6). At time-2 and time-3, there was no intervention effect for total falls in the post hoc subgroup analysis stratified by baseline dementia severity (online supplemental table 7).

#### Hospital transfers and admissions

There were no statistically significant differences between the groups for emergency department (ED) visits, ED and admission or admissions only at either time-2 or time-3. This was also the case in the post hoc analysis by dementia severity, although the numbers of admissions only at time-2 for both levels of dementia severity and at time-3 for stage ≥5 were so low that statistical tests were not done (online supplemental table 8, 9).

#### RAC admissions

There were no statistically significant differences between the groups for RAC admissions. Notably, in post hoc analysis, by time-3, the intervention group had substantially smaller proportion of mild dementia clients admitted to RAC than moderate/severe dementia (3% vs 39%), while for the control group, the proportions of admissions for mild and moderate/severe dementia were similar (18% vs 21%). However, no group difference in RAC entry was found either in mild or in moderate/severe dementia (see table 4).

**Table 4:** Residential aged care admission at time-2 (4 months) and time-3 (12 months) and by dementia severity

Dementia severity	Intervention	Control
Time-1 to time-2 (total) Mild dementia (GDRS 4) Moderate/severe dementia (GDRS>5)	5/58 (9%) 1/32 (3%) 4/26 (15%)	2/58 (3%) 1/34 (3%) 1/24 (4%)
Time-1 to time-3 (total) Mild dementia (GDRS 4) Moderate/severe dementia (GDRS>5)	11/58 (19%) 1/32 (3%) 10/26 (39%)	11/58 (19%) 6/34 (18%) 5/24 (21%)

GDRS, Global Deterioration Rating Scale for Assessment of Primary Degenerative Dementia.

### Economic outcomes

As shown in table 5, excluding the total cost of the I-HARP intervention ($A4311.68 average/mean cost per person, APP), overall health-related client costs over 12 months for the intervention group ($A12 599.94 APP) were $A2207.28 less than control ($A14 807.22 APP). This difference would have been greater if allied health service use had been included in the cost evaluation.^26^ While the number of RAC admissions was the same between the groups, the number of days in RAC was lower for I-HARP, suggesting a protective effect and delayed admission. Compared with control, clients in the intervention group had increased utility between time-1 and time-2, which declined at a slower rate between time-2 and time-3 (table 2), suggesting I-HARP’s sustained positive impact on clients’ quality of life. Using bootstrapped estimates of cost and QALY derived from imputed data (n=20 000), the ICER for I-HARP was $A83 165.79 per QALY (effect difference 0.026; 95% CI −0.034, 0.085). Due to a high degree of uncertainty around estimates, no clear conclusion could be reached on the cost-effectiveness of I-HARP. This was also the case in the post hoc analysis by dementia severity. Notably, the overall health-related costs for clients with mild dementia were substantially less, $A7097.98 APP, in the intervention group ($A6836.59 APP) than control ($A13 934.57 APP), with a significant saving from delayed/prevented RAC entries. Details of the economic evaluation have been reported.^26^

**Table 5:** Resource use comparison

Resources			I-HARP (Intervention) n=58			Usual care (control) n=58		
Resource type	Unit	Unit cost	Usage (n)	Cost	Avg p.p	Usage (n)	Cost	Avg p.p
Respite	$ per day	$102.54	267 (6)	$27 378.18	$472.04	129 (5)	$13 277.66	$228.06
Specialist	Consult	$95.00	370 (54)	$35 150.00	$606.03	379 (50)	$36 005.00	$620.78
General practitioner	Consult	$80.10	734 (57)	$58 793.40	$1013.68	739 (57)	$59 193.90	$1020.58
Emergency department	$ per transfer	$561.00	26 (15)	$14 586.00	$251.48	32 (18)	$17 952.00	$309.52
Medication	Total cost	Var (ind)	Var. (58)	$106 466.62	$1835.63	Var. (58)	$86 578.36	$1492.73
Hospital	$ per day	$930.00	176 (16)	$163 680.00	$2822.07	224 (17)	$207 855.00	$3583.71
Residential aged care home	$ per day	$255.10	1273 (11)	$324 742.30	$5599.01	1717 (11)	$438 006.70	$7551.84
Allied Health Service (AHS)	Consult	N/A	503 (55)	EXC	EXC	555 (48)	EXC	EXC
$Total costs excluding Intervention costs			All	$730 796.50	$12 599.94	All	$858 868.62	$14 807.22
Intervention costs*	$Total cost	Var (ind)	All (58)	$250 077.29	$4311.68	0 (0)	N/A	N/A
$Total costs including intervention costs			All	$980 873.79	$16 911.62	All	$858 868.62	$14 807.22
Subgroup of clients with mild dementia only			I-HARP (intervention) n=32			Usual care (control) n=34		
Respite	$ per day	$102.54	42 (1)	$4306.68	$134.59	85 (4)	$8715.90	$256.35
Specialist	Consult	$95.00	247 (31)	$23 465.00	$733.28	261 (30)	$24 795.00	$729.27
General practitioner	Consult	$80.10	435 (32)	$34 843.50	$1088.86	491 (34)	$39 329.10	$1156.74
Emergency department	$ per transfer	$561.00	12 (8)	$6732.00	$210.38	16 (9)	$8976.00	$264
Medication	Total cost	Var (ind)	Var. (32)	$57 560.00	$1798.75	Var. (58)	$55 819.66	$1641.75
Hospital	$ per day	$930.00	90 (7)	$83 700	$2615.63	102.5 (11)	$95 325.00	$2803.68
Residential aged care home	$ per day	$255.10	32 (1)	$8163.20	$255.10	944 (6)	$240 814.40	$7082.78
$Total costs excluding intervention costs				$218 770.38	$6836.59	All	$473 775.06	$13 934.57
Intervention costs*	$Total cost	Var (ind)	All (32)	$142 937.79	$4466.81	0 (0)	N/A	N/A
$Total costs including intervention costs			All	$361 708.17	$11 303.40	All	$473 775.06	$13 934.57

All costs reported in Australian dollars ($A) indexed to 2022. Costs from a health system perspective are sourced from Medicare MBS (respite, specialist visits, GP visits) as at 2022. Allied health service costs cannot be reliably calculated from collected data and so are EXC from cost analysis. Where multiple service types are listed, the average cost of the list is applied (eg, GP visit short, medium or long, medium visit price is applied). Residential aged care costs from the previous report^41^ and adjusted to 2018/2019 financial year. Individual client and intervention costs were collected within the trial period.

* Intervention costs include clinician time (RN, OT, CC) ($2573.57); home modification/assistive device ($440.85); other AHS Costs ($54.22); travel (mileage) ($339.67) and staff training ($903.36).

Avg p.p, average cost of resource per person over the whole trial; CC, case coordinator; EXC, excluded; GP, general practitioner; I-HARP, Interdisciplinary Home-bAsed Reablement Programme; N/A, not available; OT, occupational therapist; RN, registered nurse.

## Discussion

I-HARP is a novel multimodal, interdisciplinary dementia-specific rehabilitation programme. It is designed to improve and maintain the person’s independence in daily functioning with a strong focus on person-directed goal-setting, an individually tailored plan of action and strategies that are designed to enhance the fit between the person and their living environment to optimise daily function. OTs and RNs conduct comprehensive assessments that holistically addresses the impact of dementia and comorbidities on the person’s daily functioning while considering the interplay between their social, psychological, clinical and emotional needs and environments. In I-HARP, a family carer receives psychoeducation and support sessions and becomes a partner in care providing support to the person with dementia throughout the programme.

Our pragmatic RCT showed no significant effect of I-HARP on functional independence, mobility, quality of life and depression of people with dementia, or carer burden and quality of life at 4 and 12 months. No significant difference was observed in hospital transfers/admissions or RAC admissions. While the I-HARP group had somewhat better mean results for most outcome measures than usual care at both time-2 and time-3, the only statistically significant intervention effect was a reduction in home environment hazards at time-2, which may have contributed to the significantly lower rate of falls in the intervention group at time-3 compared with controls: 93 total falls by 33 intervention clients vs 252 falls by 33 control clients.

The overall results of I-HARP are in contrast to those of some other RCTs of rehabilitation programmes which successfully led to improved client functional independence and/or carer well-being.^2^ For example, the COPE (Care of Person with Dementia in their Environments) programme,^27^ the Dutch community-based OT programme^28^ and a UK-based cognitive rehabilitation intervention^29^ focus on mild to moderate dementia, consider the person’s capabilities, and physical and social environments, and work with carers through individually tailored approaches, goals and action-planning strategies based on comprehensive assessments. While I-HARP and the COPE programme involved both OTs and RNs to address clients’ underlying medical conditions and home visits over 4 months, the Dutch programme and cognitive rehabilitation were led solely by OTs over 5 weeks and 8 weeks, respectively. Potential explanations for the lack of significant effectiveness of I-HARP might include the I-HARP’s longer interval of 4–6 months, following the stop-the-clock rule, between the baseline and postintervention assessments, compared with other programmes’ 3–4 months interval. Furthermore, while most I-HARP pilot clients had mild dementia, 43% of the main I-HARP trial clients had moderate to severe dementia.

Our I-HARP qualitative study suggests that dementia severity is a major barrier to the implementation of client goals and action-planning strategies.^30^ Alternative rehabilitation approaches may be more suitable for those with greater dementia severity. Post hoc subgroup analysis supports this as I-HARP appeared to be effective in enhancing functional independence of people with mild dementia in the short term and slowing down the rate of functional decline longer term. Both the I-HARP pilot^13^ and this main trial of the subgroup with mild dementia show a consistent outcome trajectory. The home environmental safety improved in the intervention group with moderate to severe dementia, although only in the short term. This change may have been influenced by four residents with moderate to severe dementia moving to RAC where environmental safety is likely to be higher than their home environment.

The subgroup analysis results emphasise the importance of timely/early dementia intervention. This contrasts with the public’s reluctance for preventive approaches to dementia care.^31 32^ During our I-HARP pilot and main trial, we encountered several eligible potential clients with mild dementia who declined study participation as they saw ‘no need’ for any intervention as they were ‘doing fine’, as well as carers who were very keen for their family member with severe dementia to participate in the trial but could not do so as they were ineligible. Low uptake of support services by people with dementia and carers in the early stages of dementia is common, often driven by individuals’ attitudes, stigma, therapeutic nihilism and misperception and a lack of knowledge and understanding about dementia and dementia care,^32^,^34^ as well as service-related factors such as accessibility, quality and relevance.^35^ This is compounded by rehabilitation being perceived as suitable for those with physical disability, while dementia is often seen as a ‘memory problem’ only, especially in the early stages.^1 36^

Another notable and often missed aspect is the relative effect of dementia rehabilitation on improving individual’s functional independence/self-care ability, compared with the pharmacological treatment effects accompanied by risks of unwanted/adverse events. The COPE programme’s success in improving functional ability of people with dementia compared favourably with antidementia drugs and was free of adverse events.^27^ The recent amyloid lowering trials of lecanemab^37^ and donanemab^38^ reported 36% and 40% less decline, respectively, in daily function at 18 months. In I-HARP, the intervention group with mild dementia had slight improvement in daily function at 4 months and 59% less decline at 12 months, compared with controls without any of the side effects observed from those amyloid lowering agents.

Cost-effectiveness was not demonstrated, but I-HARP’s sustained positive impact on clients’ quality of life and functional independence, although only for those with mild dementia, combined with a lower resource use, are highly promising. The I-HARP’s protective effect and delayed RAC admission also signal potential cost savings for many developed countries, aligned with their government policy goal of delaying or preventing RAC admission.^39^ Further research is warranted to confirm the cost-effectiveness of the I-HARP model for people in the early stages of dementia.

We did not achieve the target sample size of 128 dyads for analysis, partly due to COVID-19. Although we hoped to recruit mainly people with mild dementia, almost half had moderate to severe dementia. This enabled post hoc analysis of these subgroups separately, but there was insufficient power to test whether the intervention effect differed in the subgroups using a test for interaction. Conclusions from these post hoc analyses should be interpreted with caution.

## Conclusion

Our 5-year pragmatic RCT of an interdisciplinary rehabilitation model did not meet its primary outcomes for a population of people with dementia, where severity ranged from mild to moderate and severe. It is suggested that I-HARP may be best suited to people in the earlier stages of dementia as a postdiagnostic support service. It provides a potential early intervention model of care in the community context, with a goal to inform the development of rehabilitation services that can be routinely offered to people living with dementia in their home. A different rehabilitation model may be required as dementia becomes more severe.

## Supplementary material

10.1136/jnnp-2024-334514 online supplemental file 1

## Data Availability

Data are available on reasonable request.
