# Supplementary material for: Effectiveness of the Interdisciplinary Home-bAsed Reablement Programme (I-HARP) on improving functional independence of people living with dementia: a multicentre, pragmatic, randomised, open-label, controlled trial
Source: J Neurol Neurosurg Psychiatry. 2025 Jan 16;96(7):e334514. doi: 10.1136/jnnp-2024-334514 (PMC12322460; doi:10.1136/jnnp-2024-334514)
Supplement: online supplemental file 1 [file jnnp-96-7-s001.docx]

**SUPPLEMENTARY INFORMATION**

**ACRONYMS**

Collateral Source version of the Geriatric Depression Scale-15 item (CS-GDS-15)

Disability Assessment for Dementia (DAD)

5-level EQ-5D version (EQ-5D-5L)

EQ-5D-5L Visual Analogue Scale (VAS)

Global Deterioration Rating Scale for Assessment of Primary Degenerative Dementia (GDRS)

Home Safety Self-Assessment Tool (HSSAT)

Interdisciplinary Home-bAsed reablement Program (I-HARP)

Quality of life in Alzheimer’s disease (QOL-AD)

Short Physical Performance Battery (SPPB)

Zarit Burden Inventory (ZBI)

**eMETHODS**

**Study procedures**

During their initial visits, I-HARP clinicians completed a comprehensive assessment relevant to their field, encompassing cognitive and functional abilities, strength, balance and home safety risks, medication regimen, pain, incontinence, depression and other chronic disease management issues, as well as the carer’s understanding of dementia, the carer’s and client’s social history and the carer’s current needs and concerns. Following an in-depth, interdisciplinary case conference, a person-centred goal setting process including the client and the carer was used to identify individualised goals and action strategies for the following 3 months. The remaining home visits of each I-HARP clinician, focussed on implementing individually tailored strategies to achieve the client’s goals, including cognitive rehabilitation, combining compensatory (e.g. calendars, diaries, reminders) and restorative strategies (e.g. mnemonics, semantic association, spaced retrieval), energy conservation and task simplification strategies, balance and strength exercises, pain relief, anxiety and depression management, problem solving, medication simplification/adherence training, and minor home alterations and assistive devices. I-HARP clinicians and case coordinators received on-going supervision and mentoring support in delivering the intervention and their performance was periodically reviewed for quality checks. Fortnightly emails and phone conversations between all clinicians occurred throughout the duration of the program.

In response to the COVID-19 pandemic public health order and following relevant best practice guidelines, a set of alternative arrangements for intervention delivery and assessments were established based on the clinician's assessment of risks and benefits such as telephone/video conferencing or the use of appropriate infection control procedures when visiting homes. Nine participants received one or two of the ten sessions via telephone/video conferencing and four of them had a 4 week stop-the-clock due to the public health order that did not allow for any home visit for two months in 2020. Assessments were also slightly delayed or completed via phone/video assessment (8 dyads for Time-2 and 7 dyads for Time-3 assessments). During the COVID-19 pandemic, following a brief cessation, ethics approval was gained to resume the intervention for existing clients as it was deemed an important part of care. However, recruitment and enrolment of new participants were severely impacted by the pandemic.

**Missing data**

Due to missing data (8.2%), three different models were run on unadjusted data (Tables S2 and S3). Model 1 was based only on observed items, and excluded clients who died or withdrew. In Model 2, handling of missing values was as per developer guidelines for the QoL-AD^1^ while for the CS-GDS-15, EQ-5D-5L and ZBI we used the half-scale rule, whereby if ≥50% of the items were answered, the participant’s mean of all non-missing items was substituted. Withdrawn/deceased participants were excluded. Model 3 proceeded as per Model 2 for the CS-GDS-15, QoL-AD, ZBI and EQ-5D-5L, with multiple imputation of all other missing values for all scales, including withdrawn clients and carers, and deceased participants were assigned the worst possible scores^2^ (relevant at Time-3 only). There was no difference in overall interpretation across the models, and results based on multiply imputed data are reported. All multiple imputations used the Markov Chain Monte Carlo (MCMC) algorithm with predictive mean matching using 5 nearest neighbours and 20 imputations.

**eRESULTS**

**Table S1: Baseline differences between those who continued to Time-2 (T-2, 4 months) and those who did not continue**

|  | **Continued to T-2**  **(n = 116)**  **Mean (SD)** | **Did not continue to T2**  **(Withdrew n = 12)**  **(Died n = 2) Mean (SD)** | **Difference, (95% CI), P value** |
| --- | --- | --- | --- |
| DAD | 59.83 (23.48) | 59.47 (24.04) | 0.36 (-12.77, 13.48), 0.96 |
| CS-GDS-15 | 6.39 (3.86) | 6.93 (4.62) | -0.54 (- 2.76, 1.67), 0.63 |
| Client QoL-AD | 35.74 (6.41) | 33.70 (5.22) | 2.04 (-1.49, 5.57), 0.26 |
| Proxy QoL-AD | 29.56 (5.73) | 29.21 (7.58) | 0.35 (- 2.98, 3.68), 0.84 |
| Client EQ5D-5L | 0.78 (0.23) | 0.67 (0.24) | 0.11 (- 0.01, 0.25), 0.07 |
| Client VAS | 73.09 (18.89) | 69.92 (15.58) | 3.46 (- 7.06, 13.97), 0.52 |
| **Carer EQ5D-5L** | **0.75 (0.23)** | **0.61 (0.32)** | **0.14 ( 0.002, 0.27), 0.047** |
| Carer VAS | 76.06 (16.79) | 69.93 (19.56) | 6.13 (- 3.45, 15.71), 0.21 |
| SPPB | 6.97 (2.73) | 5.71 (2.73) | 1.26 (- 0.27, 2.79), 0.11 |
| HSSAT | 15.11 (5.29) | 15.29 (7.00) | -0.18 (- 3.25, 2.90), 0.91 |
| ZBI | 30.82 (13.48) | 32.50 (13.25) | -1.68 (-9.22, 5.87), 0.66 |
| ACE | 53.94 (19.87) | 56.29 (10.87) | -2.34 (-9.42, 4.75), 0.50 |

Disability Assessment for Dementia (DAD), Collateral Source version of the Geriatric Depression Scale-15 item (CS-GDS-15), Quality of life in Alzheimer’s disease (QOL-AD), 5-level EQ-5D version (EQ-5D-5L), EQ-5D-5L Visual Analogue Scale (VAS), Short Physical Performance Battery (SPPB), Home Safety Self-Assessment Tool (HSSAT), Zarit Burden Inventory (ZBI)

**Table S2: Sensitivity primary analyses - differences baseline to Time-2 (4 months)**

|  | | **Model 1**  **Difference Intervention – Control* (95% CI), *P value*** | **Model 2**  **Difference Intervention - Control* (95% CI), *P value*** | **Model 3 – used for reporting all outcomes**  **Difference Intervention - Control* (95% CI), *P value*** |
| --- | --- | --- | --- | --- |
| DAD | | 3.54 (-3.32, 10.41), *0.31* | 3.54 (-3.32, 10.41), *0.31* | 3.54 (-3.32, 10.41), *0.31* |
| CS-GDS-15 | | -0.01 (-1.21, 1.18,) *0.98* | -0.31 (-1.57, 0.96), *0.63* | -0.06 (-1.35, 1.23), *0.93* |
| QOL-AD Client | | 0.10 (-2.45, 2.64), *0.94* | 0.55 (-1.34, 2.43), *0.57* | 0.87 (-1.16, 2.87), *0.40* |
| QOL-AD Proxy | | 0.57 (-1.31, 2.45) ,*0.55* | 0.05 (-1.88, 1.89), *0.96* | 0.30 (-1.57, 2.17), *0.75* |
| SPPB | | -0.24 (-1.17, 0.69), *0.61* | -0.24 (-1.17, 0.69), *0.61* | -0.03 (-1.05, 1.00), *0.96* |
| HSSAT | | -2.38 (-4.15, -0.60), *0.009* | -2.38 (-4.15, -0.60), *0.009* | -2.29 (-4.08, -0.52), *0.01* |
| ZBI | | 0.62 (-2.90, 4.14), *0.73* | 0.75 (-2.68, 4.18,), *0.67* | 0.48 (-2.96, 3.92), *0.79* |
| EQ5D5L Client  Utility score | | 0.04 (-0.03, 0.11), *0.31* | 0.04 (-0.04,0.12), *0.31* | 0.04 (-0.04, 0.14), *0.30* |
| EQ5D5L Carer  Utility score | | -0.04 (-0.12, 0.05) *0.37* | -0.05 (-0.13,0.04), *0.29* | -0.05 (-0.13, 0.05), *0.37* |
| EQ5D5L Client VAS | | -3.81 (-13.34, 5.72), *0.43* | -3.81 (-13.34, 5.72), *0.43* | -3.12 (-12.32, 6.09), *0.51* |
| EQ5D5L Carer VAS | | -2.74 (-9.23, 3.75), *0.41* | -2.74 (-9.23, 3.75,), *0.40* | -2.94 (-9.38, 3.28), *0.37* |
| When Difference is positive, Intervention decreased by less or increased by more than Control; When difference is negative, Intervention decreased more or increased by less than Control | | | | |
| Method of handling missing data | No mean substitution or multiple imputation, total scores based on valid items only, withdrawn and died excluded | | If a case had at least 11/13 QoL-AD valid obs total score calculated; if at least 50% valid obs CS-GDS-15, EQ and ZBI, then missing values replaced with that case’s mean value for the measure.  If a case had <11/13 valid obs for QoL-AD and <50% valid obs for CS-GDS-15, ZBI and EQ they are dropped from the analysis therefore sample size can be LESS in this model. | As per Model 2 plus multiple imputation has been used for QoL-AD when <11/13 valid observations, for CS-GDS-15, EQ-5D-5L and ZBI when <50% valid observations and for all other measures, regardless of the amount of missing data. |
| Sample sizes  C = Control  I = Intervention | Sample sizes:  DAD: C=58; I=58  GDS: C=56, I=57  CLIENT QoL-AD: C=51, I=49  PROXY QoL-AD: C=56, I=58  SPPB: C=45, I=48  HSSAT: C=56, I=53  ZBI: C=56, I=57  CLIENT EQ: C=55, I=53  CARER EQ: C=56, I=57  CLIENT VAS: C=45, I=40  CARER VAS: C=56, I=57 | | Sample sizes:  DAD: C=58; I=58  CS-GDS-15: C=53, I=56  CLIENT QoL-AD: C=49, I=46  PROXY QoL-AD: C=52, I=57  SPPB: C=45, I=48  HSSAT: C=56, I=53  ZBI: C=56, I=57  CLIENT EQ: C=55, I=53  CARER EQ: C=56, I=57  CLIENT VAS: C=45, I=40  CARER VAS: C=56, I=57 | Sample sizes:  DAD: C=58; I=58  CS-GDS-15: C=58; I=58  CLIENT QoL-AD: C=58; I=58 PROXY QoL-AD: C=58; I=58  SPPB: C=58; I=58  HSSAT: C=58; I=58  ZBI: C=58; I=58  CLIENT EQ: C=58; I=58  CARER EQ: C=58; I=58 CLIENT VAS: C=58; I=58  CARER VAS: C=58; I=58 |

Disability Assessment for Dementia (DAD), Collateral Source version of the Geriatric Depression Scale-15 item (CS-GDS-15), Quality of life in Alzheimer’s disease (QOL-AD), 5-level EQ-5D version (EQ-5D-5L), EQ-5D-5L Visual Analogue Scale (VAS), Short Physical Performance Battery (SPPB), Home Safety Self-Assessment Tool (HSSAT), Zarit Burden Inventory (ZBI)

**Table S3: Sensitivity primary analyses - differences baseline to Time-3 (12 months)**

| **Characteristic** | **Model 1**  **Difference Intervention – Control^#^ (95% CI), *P value*** | | **Model 2**  **Difference Intervention – Control^#^ (95% CI), *P value*** | **Model 3 – used for reporting all outcomes**  **Difference Intervention – Control^#^ (95% CI), *P value*** |
| --- | --- | --- | --- | --- |
| DAD Proxy | 3.50 (-4.12, 11.14), *0.36* | | 3.50 (-4.12, 11.14), *0.36* | 3.51 (-4.64, 11.66), *0.40* |
| CS-GDS-15 | -0.68 (-1.97, 0.62), *0.30* | | -0.25 (-1.67, 1.16), *0.72* | -0.78 (-2.45, 0.88), *0.35* |
| QoL-AD Client | 0.09 (-3.61, 3.78), *0.96* | | 0.77 (-1.55, 3.10), *0.51* | 2.05 (- 1.81, 5.90), *0.30* |
| QoL-AD Proxy | -0.12 (-2.23, 1.99), *0.91* | | 0.16 (-1.92, 2.24), *0.88* | 0.32 (-2.02, 2.67 ), *0.79* |
| SPPB | 0.48 (-0.81, 1.78), *0.46* | | 0.48 (-0.81, 1.78), *0.46* | 0.96 (-0.34, 2.28), *0.15* |
| **HSSAT** | 0.14 (-2.15, 2.44), *0.90* | | 0.14 (-2.15, 2.44), *0.90* | -1.76 (-6.47, 2.93), *0.46* |
| ZBI | -0.55 (-4.66, 3.55), *0.79* | | -0.72 (-4.61, 3.18), *0.72* | -1.85 ( -7.44, 3.74), *0.52* |
| EQ5D5L Client  Utility score | 0.06 (-0.04, 0.17), *0.23* | | 0.06 (-0.04, 0.17), *0.22* | 0.06 (-0.05, 0.18), *0.25* |
| EQ5D5L Carer  Utility score | -0.01 (-0.11, 0.09), *0.84* | | -0.003 (-0.10, 0.10), *0.95* | 0.05 (-0.07, 0.14), *0.51* |
| EQ5D5L Client VAS | -1.53 (-10.93, 7.85), *0.75* | | -1.53 (-10.93, 7.85), *0.75* | 1.95 (-8.31, 12.19), *0.71* |
| EQ5D5L Carer VAS | 0.80 (-7.36, 8.96), *0.85* | | 0.80 (-7.36, 8.96), *0.85* | 3.63 (-5.26, 12.51), *0.42* |
| ^#^ When Difference is positive, Intervention decreased by less or increased by more than Control; When difference is negative, Intervention decreased more or increased by less than Control | | | | |
| Method of handling missing data | No mean substitution or multiple imputation, total scores based on valid items only, withdrawn and died excluded. | If a case had at least 11/13 QoL-AD valid obs total score calculated; if at least 50% valid obs CS-GDS-15, EQ and ZBI, then missing values replaced with that case’s mean value for the measure.  If a case had <11/13 valid obs for QoL-AD and <50% valid obs for CS-GDS-15, ZBI and EQ they are dropped from the analysis therefore sample size can be LESS in this model. | | As per Model 2 plus multiple imputation has been used for QoL-AD when <11/13 valid observations, for CS-GDS-15, EQ-5D-5L and ZBI when <50% valid observations and for all other measures, regardless of the amount of missing. Worst possible scores for all outcomes used for participants who died. |
|  | Sample sizes:  DAD: C1=54, I=53  CS-GDS-15: C=53, I=51  CLIENT QoL-AD: C=45, I=40  PROXY QoL-AD:C=54, I=52  SPPB: C=40, I=32  HSSAT: C=48, I=43  ZBI: C=52, I=51  CLIENT EQ: C=51,I=46  CARER EQ: C=51, I=52  CLIENT VAS: C=40,I=34  CARER VAS:C=51, GI=52 | Sample sizes:  DAD: C=54, I=53  CS-GDS-15:C=48, I=44  CLIENT QoL-AD: C=36, I=33  PROXY QoL-AD:C=51, I=50  SPPB: C=40, I=32  HSSAT: C=48, I=43  ZBI:C=51, I=51  CLIENT EQ:C=51, I=46  CARER EQ: c=51, I=52  CLIENT VAS: C=40, I=34  CARER VAS: C=51, I=52 | | Sample sizes:  DAD: C=58, I=58  CS-GDS-15: C=58, I=58  CLIENT QoL-AD: C=58, I=58 PROXY QoL-AD: C=58, I=58  SPPB: C=58, I=58  HSSAT: C=58, I=58  ZBI: C=58, I=58  CLIENT EQ: C=58, I=58  CARER EQ: C=58, I=58 CLIENT VAS: C=58, I=58  CARER VAS: C=58, I=58 |

Disability Assessment for Dementia (DAD), Collateral Source version of the Geriatric Depression Scale-15 item (CS-GDS-15), Quality of life in Alzheimer’s disease (QOL-AD), 5-level EQ-5D version (EQ-5D-5L), EQ-5D-5L Visual Analogue Scale (VAS), Short Physical Performance Battery (SPPB), Home Safety Self-Assessment Tool (HSSAT), Zarit Burden Inventory (ZBI)

**Table S4: Post-hoc subgroup analysis based on dementia severity at baseline: Comparisons Time-1 (baseline) – Time-2 (4 months). GDRS Stage 4: Mild dementia at baseline; GDRS Stage** ≥**5: Moderate-severe dementia at baseline.**

| **Control: Stage 4 n = 34; Stage ≥5 n = 24** | | | | **Intervention: Stage 4 n = 32; Stage ≥5 n = 26** | | | |
| --- | --- | --- | --- | --- | --- | --- | --- |
| **Characteristic** | **Baseline**  **Mean (SE)** | **T2**  **Mean (SE)** | **Change (SE)** | **Baseline**  **Mean (SE)** | **T2**  **Mean (SE)** | **Change (SE)** | **Adjusted Difference between Group 1 and Group 2^**  **(95% CI)** |
| **Stage 4: DAD** | **73.33 (3.16)** | **62.39 (4.24)** | **-10.94 (3.14)** | **66.32 (3.34)** | **66.61 (3.05)** | **0.29 (2.45)** | **8.99 (1.21, 16.79)^*^** |
| Stage ≥5: DAD | 42.97 (4.23) | 34.99 (4.80) | - 7.98 (4.14) | 49.74 (4.67) | 35.63 (5.42) | -14.11 (3.95) | -4.39 (-15.61, 6.86)**^*^** |
| Stage 4: CS-GDS-15 | 5.77 (0.63) | 5.82 (0.61) | 0.05 (0.51) | 6.35 (0.75) | 6.14 (0.75) | -0.21 (0.52) | -0.10 (-1.47, 1.28)**^*^** |
| Stage ≥5: CS-GDS-15 | 8.01 (0.92) | 7.02 (0.86) | -0.99 (0.90) | 9.34 (0.82) | 8.63 (0.89) | -0.71 (0.78) | 0.66 (-1.39, 2.70)**^*^** |
| Stage 4: Client QoL-AD | 37.52 (0.83) | 35.74 (0.93) | -1.78 (0.81) | 36.28 (0.95) | 36.48 (1.00) | 0.20 (0.88) | 1.77 (-0.44, 3.97)**^*^** |
| Stage ≥5: Client QoL-AD | 36.81 (1.00) | 36.69 (1.24) | -0.12 (1.10) | 37.49 (1.25) | 36.71 (1.25) | -0.78 (1.37) | -0.40 (-3.69, 2.90)**^*^** |
| Stage 4: Proxy QoL-AD | 31.83 (1.00) | 31.15 (1.07) | -0.69 (0.80) | 31.97 (0.91) | 32.62 (0.94) | 0.65 (0.80) | 1.53 (-0.63, 3.63)**^*^** |
| Stage ≥5:Proxy QoL-AD | 27.29 (0.92) | 28.81 (0.90) | 1.52 (1.04) | 29.36 (1.08) | 29.73 (1.07) | 0.38 (1.23) | 0.41 (-2.25, 3.07)**^*^** |
| Stage 4: SPPB | 7.74 (0.46) | 6.75 (0.60) | -0.99 (0.54) | 6.75 (0.54) | 6.44 (0.62) | -0.31 (0.44) | 0.51 (-0.80, 1.83)**^*^** |
| Stage ≥5: SPPB | 5.54 (0.68) | 5.00 (0.62) | -0.54 (0.52) | 6.77 (0.645 | 5.30 (0.69) | -1.47 (0.61) | -0.64 (-2.08, 0.81)**^*^** |
| Stage 4: HSSAT | 14.59 (0.89) | 12.96 (0.89) | -1.63 (0.68) | 14.69 (0.92) | 11.81 (0.82) | -2.87 (0.88) | -1.49 (-3.35, 0.37)**^*^** |
| **Stage** ≥**5: HSSAT** | **14.83 (1.02)** | **12.38 (1.18)** | **-2.45 (0.92)** | **16.58 (1.02)** | **10.60 (1.16)** | **-5.98 (1.15)** | **-3.14 (-6.10, -0.17)^*^** |
| Stage 4: ZBI | 27.80 (2.55) | 30.38 (2.52) | 2.58 (1.66) | 27.42 (2.38) | 28.85 (2.41) | 1.43 (1.39) | -0.99 (-5.18, 3.20)*^+^* |
| Stage ≥5: ZBI | 37.20 (2.17) | 35.77 (1.83) | -1.43 (1.86) | 34.00 (2.50) | 35.36 (2.76) | 1.36 (2.19) | 1.61 (-3.71, 86.93)*^+^* |
| Stage 4: Client EQ | 0.79 (0.04) | 0.80 (0.05) | 0.01 (0.03) | 0.71 (0.04) | 0.79 (0.05) | 0.08 (0.04) | 0.05 (-0.06, 0.15)**^*^** |
| Stage ≥5: Client EQ | 0.77 (0.05) | 0.75 (0.07) | -0.02 (0.06) | 0.86 (0.03) | 0.86 (0.03) | 0.007 (0.05) | 0.06 (-0.08, 0.20)**^*^** |
| Stage 4: Carer EQ | 0.75 (0.04) | 0.74 (0.04) | -0.01 (0.03) | 0.74 (0.05) | 0.69 (0.05) | -0.05 (0.05) | -0.04 (-0.15, 0.07)*^+^* |
| Stage ≥5: Carer EQ | 0.73 (0.04) | 0.66 (0.06) | -0.07 (0.05) | 0.75 (0.04) | 0.65 (0.06) | -0.10 (0.04) | -0.03 (-0.16, 0.10)*^+^* |
| Stage 4: Client VAS | 77.25 (2.99) | 76.36 (2.80) | -0.89 (3.82) | 72.96 (2.93) | 71.37 (2.95) | -1.59 (3.67) | -3.88 (-12.02, 4.26)**^*^** |
| Stage ≥5: Client VAS | 68.59 (4.50) | 74.91 (5.01) | 6.32 (6.90) | 70.11 (4.90) | 69.79 (4.80) | -0.32 (6.56) | -5.82 (-18.69, 7.05)*^*^* |
| Stage 4: Carer VAS | 75.56 (2.95) | 77.12 (2.60) | 1.56 (2.21) | 76.34 (3.44) | 75.13 (3.43) | -1.21 (3.60) | -1.47 (-8.78, 5.84)*^+^* |
| Stage ≥5: Carer VAS | 76.70 (2.94) | 74.54 (3.63) | -2.16 (3.22) | 75.23 (3.28) | 70.19 (3.52) | -5.04 (4.13) | -3.57 (-12.90, 75.77)*^+^* |

^ Difference indicates how much higher (if positive) or lower (if negative) Intervention mean score is vs Control mean score in adjusted analysis

* Adjusted for Site, Dementia Severity, Client Pension, Client Age and baseline score of the outcome; + Adjusted for Site, Dementia Severity, Carer Age, Carer Pension and baseline score

**Table S5. Post-hoc subgroup analysis based on dementia severity at baseline: Comparisons Time-1 (baseline) – Time-3 (12 months). GDRS Stage 4: Mild dementia at baseline; GDRS Stage ≥5: Moderate-severe dementia at baseline.**

| **Control**  **Stage 4 n = 34**  **Stage ≥5 n = 24** | | | | **Intervention**  **Stage 4 n = 32**  **Stage ≥5 n = 26** | | | |
| --- | --- | --- | --- | --- | --- | --- | --- |
| **Characteristic** | **Baseline**  **Mean (SE)** | **T3**  **Mean (SE)** | **Change (SE)** | **Baseline**  **Mean (SE)** | **T3**  **Mean (SE)** | **Change (SE)** | **Adjusted Difference between Group 1 and Group 2^ (95% CI)** |
| **Stage 4: DAD** | **73.33 (3.16)** | **50.00 (4.75)** | **-23.33 (3.73)** | **66.32 (3.34)** | **56.69 (4.09)** | **-9.64 (3.44)** | **12.16 (1.93, 22.38)^*^** |
| Stage ≥5: DAD | 42.97 (4.23) | 36.32 (4.53) | - 6.65 (4.57) | 49.74 (4.67) | 32.81 (5.22) | -16.93 (4.64) | - 7.16 (-18.78, 4.46)^*^ |
| Stage 4: CS-GDS-15 | 5.77 (0.63) | 7.52 (0.77) | 1.75 (0.76) | 6.35 (0.75) | 7.11 (0.78) | 0.76 (0.62) | -0.87 (-2.741.01)^*^ |
| Stage ≥5: CS-GDS-15 | 8.01 (0.92) | 7.82 (0.89) | -0.19 (0.98) | 9.34 (0.82) | 8.77 (0.98)9 | -0.57 (1.02) | 0.69 (-1.77, 3.14)^*^ |
| Stage 4: Client QoL-AD | 37.52 (0.83) | 35.36 (1.71) | -2.16 (1.55) | 36.28 (0.95) | 36.16 (1.40) | -0.12 (1.27) | 1.66 (-2.32, 5.65)^*^ |
| Stage ≥5: Client QoL-AD | 36.81 (1.00) | 32.96 (2.24) | -3.85 (2.08) | 37.49 (1.25) | 35.82 (2.27) | -1.66 (2.39) | 2.14 (-3.79, 8.06)^*^ |
| Stage 4: Proxy QoL-AD | 31.83 (1.00) | 30.05 (1.17) | -1.78 (1.17) | 31.97 (0.91) | 31.65 (1.05) | -0.32 (1.02) | 1.43 (-1.43, 4.29)^*^ |
| Stage ≥5: Proxy QoL-AD | 27.29 (0.92) | 28.16 (1.22) | 0.87 (1.19) | 29.36 (1.08) | 28.93 (1.46) | -0.43 (1.43) | -0.78 (-4.20, 2.64)^*^ |
| Stage 4: SPPB | 7.74 (0.46) | 5.46 (0.67) | -2.28 (0.62) | 6.75 (0.54) | 5.90 (0.67) | -0.85 (0.57) | 1.05 (-0.64, 2.73)^*^ |
| Stage ≥5: SPPB | 5.54 (0.68) | 3.99 (0.70) | -1.55 (0.58) | 6.77 (0.45) | 5.58 (1.01) | -1.19 (0.93) | 0.96 (-1.27, 3.20)^*^ |
| Stage 4: HSSAT | 14.59 (0.89) | 13.32 (2.31) | -1.27 (2.34) | 14.69 (0.92) | 12.60 (1.45) | -2.09 (1.35) | -0.45 (-5.53, 4.63)^*^ |
| Stage ≥5: HSSAT | 14.83 (1.17) | 12.70 (3.00) | -2.13 (3.18) | 16.58 (1.02) | 11.57 (2.94) | -5.01 (3.12) | -051 (-8.50, 7.47)^*^ |
| Stage 4: ZBI | 27.80 (2.55) | 33.63 (3.04) | 5.82 (2.32) | 27.42 (2.38) | 30.13 (2.71) | 2.71 (1.89) | -1.77 (-7.634.08)^+^ |
| Stage ≥5: ZBI | 37.20 (2.17) | 38.35 (3.45)3 | 1.15 (3.66) | 34.01 (2.51) | 35.23 (3.59) | 1.22 (3.73) | -1.82 (-11.74, 108.10)^+^ |
| Stage 4: Client EQ | 0.79 (0.04) | 0.72 (0.04) | -0.06 (0.05) | 0.71 (0.04) | 0.74 (0.04) | 0.03 (0.05) | 0.04 (-0.09, 0.17)^*^ |
| Stage ≥5: Client EQ | 0.77 (0.05) | 0.66 (0.06) | -0.11 (0.06) | 0.86 (0.03) | 0.78 (0.05) | -0.08 (0.06) | 0.07 (-0.08, 0.22)^*^ |
| Stage 4: Carer EQ | 0.75 (0.04) | 0.73 (0.04) | -0.02 (0.05) | 0.74 (0.05) | 0.74 (0.05) | 0.004 (0.06) | 0.02 (-0.10, 0.14)^+^ |
| Stage ≥5: Carer EQ | 0.73 (0.05) | 0.62 (0.07) | -0.11 (0.06) | 0.75 (0.04) | 0.71 (0.05) | -0.04 (0.05) | 0.07 (-0.09, 0.23)^+^ |
| Stage 4: Client VAS | 77.25 (2.99) | 71.17 (3.89) | -6.08 (4.02) | 72.96 (2.93) | 72.24 (3.94) | -0.72 (3.92) | 3.43 (-6.72, 13.58)^*^ |
| Stage ≥5: Client VAS | 68.59 (4.50) | 66.68 (5.59) | -1.90 (6.77) | 70.11 (4.90) | 65.61 (5.80) | -4.49 (6.77) | -2.76 (-18.39, 12.88)^*^ |
| Stage 4: Carer VAS | 75.56 (2.95) | 72.50 (3.30) | -3.06 (3.81) | 76.34 (3.44) | 75.65 (3.01) | -0.68 (3.84) | 3.13 (-5.73, 11.98)^+^ |
| Stage ≥5: Carer VAS | 76.70 (2.94) | 63.90 (4.92) | -12.80 (4.66^)^ | 75.23 (3.28) | 68.36 (4.92) | -6.87 (6.00) | 5.00 (-8.78, 18.79)^+^ |

^ Difference indicates how much higher (if positive) or lower (if negative) Intervention mean score is vs Control mean score in adjusted analysis

* Adjusted for Site, Dementia Severity, Client Pension, Client Age and baseline score of the outcome; + Adjusted for Site, Dementia Severity, Carer Age, Carer Pension and baseline score

**Table S6 Prevalence and outcomes of falls at Time-2 (4 months) and Time-3 (12 months) in intervention and control groups.**

|  | **Exp (B)** | **95% CI** | **P value** | **Number of falls / sample size** | **Mean number falls*** |
| --- | --- | --- | --- | --- | --- |
| Total falls Time-2^*^  Total falls Time-3^*^ | 0.54  0.40 | 0.25, 1.19  0.21, 0.75 | 0.13  **0.005** | Intervention: 46/58; Control: 81/58  Intervention: 93/55; Control: 252/56 | Intervention: 0.76; Control: 1.41  Intervention: 1.76; Control: 4.42 |
| Falls with minor injury Time-2^*^  Falls with minor injury Time-3^*^ | 0.71  0.48 | 0.26, 1.97  0.23, 1.00 | 0.51  0.05 | Intervention: 27/58; Control: 38/58  Intervention: 53/55; Control: 114/56 | Intervention: 0.45; Control: 0.63  Intervention: 0.98; Control: 2.05 |
| Falls with major injury Time-2^*^  Falls with major injury Time-3^*^ | 0.62  0.65 | 0.23, 1.73  0.34, 1.24 | 0.36  0.19 | Intervention: 6/58; Control: 10/58  Intervention: 15/55; Control: 24/56 | Intervention: 0.08; Control: 0.13  Intervention: 0.30; Control: 0.47 |
| Falls without injury Time-2^*^  Falls without injury Time-3^*^ | 0.65  0.48 | 0.12, 3.49  0.17, 1.39 | 0.62  0.18 | Intervention: 13/58; Control: 33/58  Intervention: 25/55; Control: 114/56 | Intervention: 0.24; Control: 0.57  Intervention: 0.50 Control: 1.05 |

* Adjusted for Site, Dementia Severity, Client Pension, Client Age

|  | **Exp (B)** | **95% CI** | **# Falls** | **Mean # falls*** |
| --- | --- | --- | --- | --- |
| Total falls Time-2^*^  GDRS Stage 4  GDRS Stage ≥5  Total falls Time-3^*^  GDRS Stage 4  GDRS Stage ≥5 | 0.67  0.85  0.43  0.87 | 0.19, 2.36  0.29, 2.49  0.16, 1.12  0.37, 2.05 | Intervention: 32/32; Control: 56/34  Intervention: 14/26; Control: 25/24  Intervention: 57/31; Control: 183/33  Intervention: 36/24; Control: 69/23 | Intervention: 1.04; Control: 1.54  Intervention: 0.61; Control: 0.72  Intervention: 2.01; Control: 4.73  Intervention: 1.85; Control: 2.13 |
| Falls with minor injury Time-2**^*^**  GDRS Stage 4  GDRS Stage ≥5  Falls with minor injury Time-3^*^  GDRS Stage 4  GDRS Stage ≥5 | 1.39  0.26  0.71  0.71 | 0..35, 5.60  0.04, 1.83  0.26, 1.98  0.21, 2.42 | Intervention: 22/32; Control: 17/23  Intervention: 5/26; Control: 21/24  Intervention: 37/31; Control: 62/33  Intervention: 16/24; Control: 52/23 | Intervention: 0.69; Control: 0.49  Intervention: 0.15; Control: 0.58  Intervention: 1.21; Control: 1.70  Intervention: 0.86; Control: 1.22 |
| Falls with major injury Time-2^*^  GDRS Stage 4  GDRS Stage ≥5  Falls with major injury Time-3^*^  GDRS Stage 4  GDRS Stage ≥5 | 0.37  1.30  0.51  0.97 | 0.06, 2.19  0.40, 4.18  0.21, 1.26  0.37, 2.57 | Intervention: 2/32; Control: 7/34  Intervention: 4/26; Control: 3/24  Intervention: 7/31; Control: 15/33  Intervention: 8/24; Control: 9/23 | Intervention: 0.06; Control: 0.15  Intervention: 0.15; Control: 0.12  Intervention: 0.25; Control: 0.49  Intervention: 0.34; Control: 0.35 |
| Falls without injury Time-2^*^  GDRS Stage 4  GDRS Stage ≥5  Falls without injury Time-3^*^  GDRS Stage 4  GDRS Stage ≥5 | 0.29  6.33  0.18  1.55 | 0.04, 2.14  0.42, 6.43  0.10, 0.33  0.63, 3.82 | Intervention: 8/32; Control: 32/34  Intervention: 5/26; Control: 1/24  Intervention: 13/31; Control: 106/33  Intervention: 12/26; Control: 8/24 | Intervention: 0.27; Control: 0.92  Intervention: 0.14; Control: 0.02  Intervention: 0.31; Control: 1.71  Intervention: 0.47; Control: 0.30 |

**Table S7 Prevalence and outcomes of falls at Time-2 (4 months) and Time-3 (12 months) in intervention and control groups; stratified by dementia**

Severity at baseline. GDRS Stage 4- mild dementia; GDRS Stage ≥5 moderate-severe dementia.

*Adjusted for Site, Client Pension, Client Age

**Table S8. Hospital transfers and admissions at Time-2 (4 months) and Time-3 (12 months).**

|  | **Exp (B)** | **95% CI** | **P value** | **# presentations and admissions** | **Mean# presentations and admissions^*^** |
| --- | --- | --- | --- | --- | --- |
| ED presentation only Time-2  ED presentation only Time-3 | 1.26  0.83 | 0.42, 3.79  0.45, 1.55 | 0.68  0.57 | Intervention: 7/58; Control:6/58  Intervention: 18/55; Control:24/56 | Intervention: 0.13; Control: 0.10  Intervention: 0.35; Control: 0.42 |
| ED presentation and Hospital admission Time-2  ED presentation and Hospital admission Time-3 | 1.85  0.80 | 0.69, 4.93  0.35, 1.85 | 0.22  0.61 | Intervention: 15/58; Control:8/58  Intervention: 17/55; Control:22/56 | Intervention: 0.24; Control: 0.13  Intervention: 0.29; Control: 0.37 |
| Admission only Time-2  Admission only Time-3 | 0.98  0.71 | 0.39, 2.50  0.17, 2.96 | 0.97  0.64 | Intervention: 3/58; Control:3/58  Intervention: 5/55; Control:6/56 | Intervention: 0.05; Control: 0.05  Intervention: 0.06; Control: 0.08 |

*Adjusted for Site, Dementia Severity, Client Pension, Client Age

**Table S9. Hospital transfers and admissions at Time-2 (4 months) and Time-3 (12 months): subgroup analysis by dementia severity at Time-1 (baseline). GDRS Stage 4 – mild dementia; GDRS Stage ≥5 = moderate to severe dementia.**

|  | **Exp (B)** | **95% CI** | **# presentations and admissions** | **Mean# presentations and admissions^*^** |
| --- | --- | --- | --- | --- |
| ED presentation only Time-2  GDRS Stage 4  GDRS Stage ≥5  ED presentation only Time-3  GDRS Stage 4  GDRS Stage ≥5 | 1.24  1.95  0.80  0.91 | 0.42, 3.60  0.61, 6.31  0.35, 1.84  0.35, 2.39 | Intervention: 3/32; Control:3/34  Intervention: 4/26; Control:3/24  Intervention: 9/31; Control:13/33  Intervention: 9/24; Control:11/23 | Intervention: 0.10; Control: 0.08  Intervention: 0.10; Control: 0.05  Intervention: 0.33; Control 0.41  Intervention: 0.38; Control 0.42 |
| ED and admission Time-2  GDRS Stage 4  GDRS Stage ≥5  ED and admission Time-3  GDRS Stage 4  GDRS Stage ≥5 | 1.52  2.22  0.66  0.86 | 0.56, 4.12  0.65, 7.55  0.20, 2.15  0.31, 2.37 | Intervention: 8/32; Control: 5/34  Intervention: 7/26; Control: 3/24  Intervention: 9/31; Control:14/33  Intervention: 8/24; Control: 8/23 | Intervention: 0.21; Control 0.14  Intervention: 0.26; Control 0.12  Intervention: 0.29; Control: 0.44  Intervention: 0.19; Control: 0.22 |
| Admission only Time-2  GDRS Stage 4  GDRS Stage ≥5  Admission only Time-3  GDRS Stage 4  GDRS Stage ≥5 | -  -  1.60  - | -  -  0.56, 4.61  - | Intervention: 3/32; Control:1/34  Intervention: 0/26; Control:2/24  Intervention: 5/31; Control:3/33  Intervention: 0/24 Control: 3/23 | -  -  Intervention: 0.14; Control: 0.09  - |

*Adjusted for Site, Client Pension, Client Age

**Supplement references**

[1] Logsdon, R. G., Gibbons, L. E., McCurry, S. M., & Teri, L. (1999). Quality of life in Alzheimer's disease: patient and caregiver reports. Journal of Mental health and Aging, 5, 21-32.
